# Development and validation of a Spanish translation of the Yale activity questionnaire

**DOI:** 10.1186/1471-2474-15-120

**Published:** 2014-04-07

**Authors:** Jeffrey N Katz, Maria T Perez, Nina N Niu, Yan Dong, Sarah A Brownlee, Scott A Elman, Derek S Stenquist, Adianez Santiago, Edward S Sanchez, Jamie E Collins

**Affiliations:** 1 Brigham and Women’s Hospital, 75 Francis Street, BC 4-016, Boston, MA 02115, USA; 2 Harvard Medical School, Boston, MA, USA; 3 Yale University School of Medicine, New Haven, CT, USA; 4 Hospital General de La Plaza de Salud, Santo Domingo, Dominican Republic; 5 Boston University School of Public Health, Boston, MA, USA

## Abstract

**Background:**

Valid measures of physical activity are critical research tools. The objective of this study was to develop a Spanish translation of the Yale Physical Activity Survey, and to provide preliminary evidence of its validity in a population of Dominican patients with lower extremity arthritis.

**Methods:**

A Dominican bilingual health care professional translated the Yale Physical Activity Survey (YPAS) from English to Spanish. Several Dominican adults reviewed the translation to ensure it was linguistically and culturally appropriate. The questionnaire was back-translated to English by a North American researcher who is fluent in Spanish. Discrepancies between the original and back-translated versions were resolved by the translator and back-translator. The Spanish translation was administered to 108 Dominican subjects with advanced hip or knee arthritis prior to (N = 44) or one to four years following (N = 64) total joint replacement. We assessed construct validity by examining the association of YPAS scores and measures of functional status and pain (WOMAC), quality of life (EQ-5D) and the number of painful lower extremity joints.

**Results:**

A higher YPAS Part II Activity Dimensions Summary Index score had weak to modest correlations with worse function and quality of life as measured with the WOMAC function scale (r = 0.21, p = 0.03), SF-36 Physical Activity Scale (r = 0.29, p = 0.004) and EQ-5D (r = 0.34, p = 0.0007). Total minutes of vigorous activity and walking had weak to modest correlation with these measures (WOMAC Function Scale (r = 0.15, p = 0.15), SF-36 Physical Activity Scale (r = 0.21, p = 0.04) and EQ-5D utility (r = 0.24, p = 0.02)). Correlations between the YPAS Part I energy expenditure score and these measures were lower (WOMAC Function Scale (r = 0.07, p = 0.49), SF-36 Physical Activity Scale (r = 0.03, p = 0.74) and EQ-5D utility (r = 0.18, p = 0.07)).

**Conclusions:**

We have developed a new Spanish translation of the Yale Physical Activity Survey and provided evidence of convergent validity in a sample of Dominican patients prior to or 1–4 years following total joint replacement.

## Background

Individuals who perform regular physical activity experience lower levels of morbidity and mortality, even after adjustment for comorbid illnesses [1-4]. Consequently, the Centers for Disease Control, World Health Organization and other public health bodies have published recommendations for regular physical activity [5,6]. Research on determinants of physical activity requires reliable, valid measures. While objective methods, such as use of accelerometry, have been validated extensively [7-9] these measures also have limitations [10,11]. Further, some research methodologies, such as the use of large surveys, require self-reported measures of physical activity. A number of such measures have been developed and validated against objective measures and subsequent health status [12-14].

The Yale Physical Activity Survey (YPAS) is one such measure [15]. The YPAS was developed to assess physical activity in community-based adults age 60 and greater. It has been shown to have acceptable intra-rater reliability and validity as compared with accelerometer data [16]. The YPAS has been used in diverse patient populations across nations and cultures [17].

We sought to measure physical activity in a cohort of Dominican patients with advanced arthritis who were about to receive a total hip or knee replacement, or who had received a hip or knee replacement one to four years prior to survey administration. Given its extensive use in ambulatory older adults, the YPAS seemed an appropriate measure. We were unable to identify a translation of the YPAS tailored for Dominican patients. Further, while at least two Spanish translations of the YPAS have been reported [17,18], we were unable to find the translated instruments, despite extensive searching and attempts to contact the authors. Thus, we undertook a translation of the YPAS that would be appropriate for patients in the Dominican Republic (DR). This paper reports on the translation process and preliminary validation of a Spanish version of the YPAS and we provide the translated instrument for interested readers and investigators.

## Methods

### Translation

Several academic and policy organizations have proposed recommendations for translating health status instruments [19-21]. These different sets of recommendations are similar to one another. We adapted the World Health Organization protocol in translating the Yale Physical Activity Survey [19]. The work was carried out by a primary translator (English to Spanish), a back-translator (Spanish to English) and a panel of investigators and lay persons from both the United States (US) and the Dominican Republic. The work proceeded in four stages: First, a native Spanish-speaking, Dominican-born nurse and medical student, who attended primary and secondary school in the Dominican Republic and college and medical school in the US, did the primary translation from English to Spanish. She attempted to identify and modify concepts that were not culturally sensitive. The translator’s work was reviewed by other bilingual Dominicans who grew up in the Dominican Republic and were not themselves health professionals. Other Dominican members of the research team provided input on the cultural sensitivity of word choice and of specific physical activities.

In the next phase, a non-Latino, English-speaking US resident, who is fluent in Spanish and had no prior knowledge of the YPAS, back-translated the measure from Spanish to English. Here too, the goal was to achieve the best possible conceptual translation, rather than literal, linguistic equivalence.

Two discrepancies between the original English YPAS and the back translation were easily resolved in a conference call that included the primary translator, back translator and several members of the research team (both Dominican and US).

Finally, the instrument was pretested by investigators, panel members and lay people in the Dominican Republic; these individuals comprised males and females and spanned a broad age range. This pilot work identified a few activities in the YPAS that the study population was unlikely to participate in (such as tennis), for either cultural or socioeconomic reasons. These were substituted for others (swimming and basketball) that are done more routinely and are similarly intensive.

### Validation

The goal of this phase was convergent validation, in which we examined the associations between YPAS scores and other measures that we hypothesized to be associated with physical activity such as functional status and self-rated health.

### Sample

The validation was fielded in the context of an annual mission trip of Operation Walk Boston, a philanthropic organization that performs approximately 45 total knee or hip replacements annually on Dominican persons with advanced hip or knee arthritis who otherwise would be unable to afford the surgery [22,23]. The host institution is Hospital General de la Plaza de la Salud in Santo Domingo, Dominican Republic. This study was approved by the Institutional Review Board of the Brigham and Women’s Hospital and Partners HealthCare. Our research has also been approved by the Ethics Committee of Hospital General de la Plaza de la Salud. Formal written consent was not required since the study involved completing questionnaires with no interventions.

Patients admitted to the hospital for a total knee or hip replacement in April 2013 completed the Spanish version YPAS, along with a battery of other health status measures, preoperatively. In addition, patients returning to the annual follow up clinic in April 2013 who had received total hip or knee replacement in 2009, 2010, 2011 or 2012, also completed the YPAS along with other measures.

### Data elements

In addition to the YPAS, the questionnaires included the WOMAC (Western Ontario MacMaster Osteoarthritis Index) Pain and Function scales [24]; the Physical Activity Scale, 5-item Mental Health Scale and a single general health item (describe your health as excellent, good, fair, poor) from the SF-36 (Short Form 36) [25]; and the Euroqol EQ-5D questionnaire [26]. We used published Spanish translations of these instruments and have documented the reliability and validity of the WOMAC and SF-36 scales in this Dominican population previously [22]. We also collected data on the number of hips and knees that were painful and on subject age.

### YPAS scoring and statistical analysis

Part I of the YPAS asks about the length of time subjects spend on specific activities in several domains (house work, yard work, care taking, exercise and recreation). These responses are aggregated to a number of minutes of each activity and then multiplied by a weight developed by the YPAS designers to yield total estimated energy expenditure. Part II asks about vigorous activities, leisurely walking, standing, moving about on one’s feet, sitting and stair climbing. This portion can be used to derive a total score (Activity Dimensions Summary Index; ADSI) as well as the number of minutes of vigorous activity and of walking. These vigorous activity and walking scores can, in turn, be used to develop a binary variable indicating whether the subject met the US Centers for Disease Control threshold of 75 minutes of vigorous activity per week or 150 minutes of vigorous or moderate activities per week, occurring in at least ten minute bouts.

We hypothesized that the total amount of activity (as represented by the YPAS Part II ADSI score) would be positively and modestly associated with greater functional status and quality of life. These hypotheses were addressed in bivariate Pearson correlation analyses. In further analyses, we hypothesized that the proportion of subjects meeting the CDC recommendations would be greater for those with better quality of life and fewer painful joints. We assessed these relationships using Fisher’s Exact test for the binary quality of life indicator and the Mantel-Haenszel test for trend for the ordinal painful joints indicator. As these validation analyses were somewhat exploratory, we accepted a critical p value of 0.05.

## Results

After the instrument had been translated and back translated, the panel made one set of changes. In Part I, the panel felt that bowling, golf and racket sports are done rarely in the DR and substituted instead fishing (for bowling), baseball or swimming (for golf), and basketball (for racket sports). The final translated instrument is shown in Additional file 1.

### Validation

The characteristics of the study sample used for the validation are shown in Table 1. In general, the population was mostly female with mean age of 61. Most had undergone or were preparing to undergo total knee (as opposed to hip) replacement.

**Table 1:** Features of the two study samples

**Feature**	**Preoperative sample (N = 44)**	**Follow-up sample (N = 64)**
Mean age (SD)	58 (14)	63 (14)
Sex		
Female (%)	36 (82%)	51 (80%)
Male (%)	8 (18%)	13 (20%)
Joint operated upon*		
Knee (%)	29 (67%)	52 (81%)
Hip (%)	14 (33%)	14 (22%)
Educational attainment		
Less than high school graduate (%)	34 (77%)	50 (81%)
High school graduate (%)	10 (23%)	12 (19%)
Number of painful joints		
0-1	15 (34%)	47 (81%)
2	21 (48%)	7 (12%)
3 or more	8 (18%)	4 (7%)
Health status measures (mean (SD))		
WOMAC** pain	55 (27)	20 (24)
WOMAC** function	60 (27)	17 (16)
SF-36 physical activity***	26 (23)	68 (25)
EQ-5D	0.56 (0.24)	0.85 (0.15)

*Percentages do not add up to 100% because one patient had both TKR and THR.

**WOMAC = Western Ontario Osteoarthritis Index; range 0–100 with 0 = best.

***Short-Form 36 Physical Activity Scale; range 0–100 with 100 = best.

**Figure 1:**
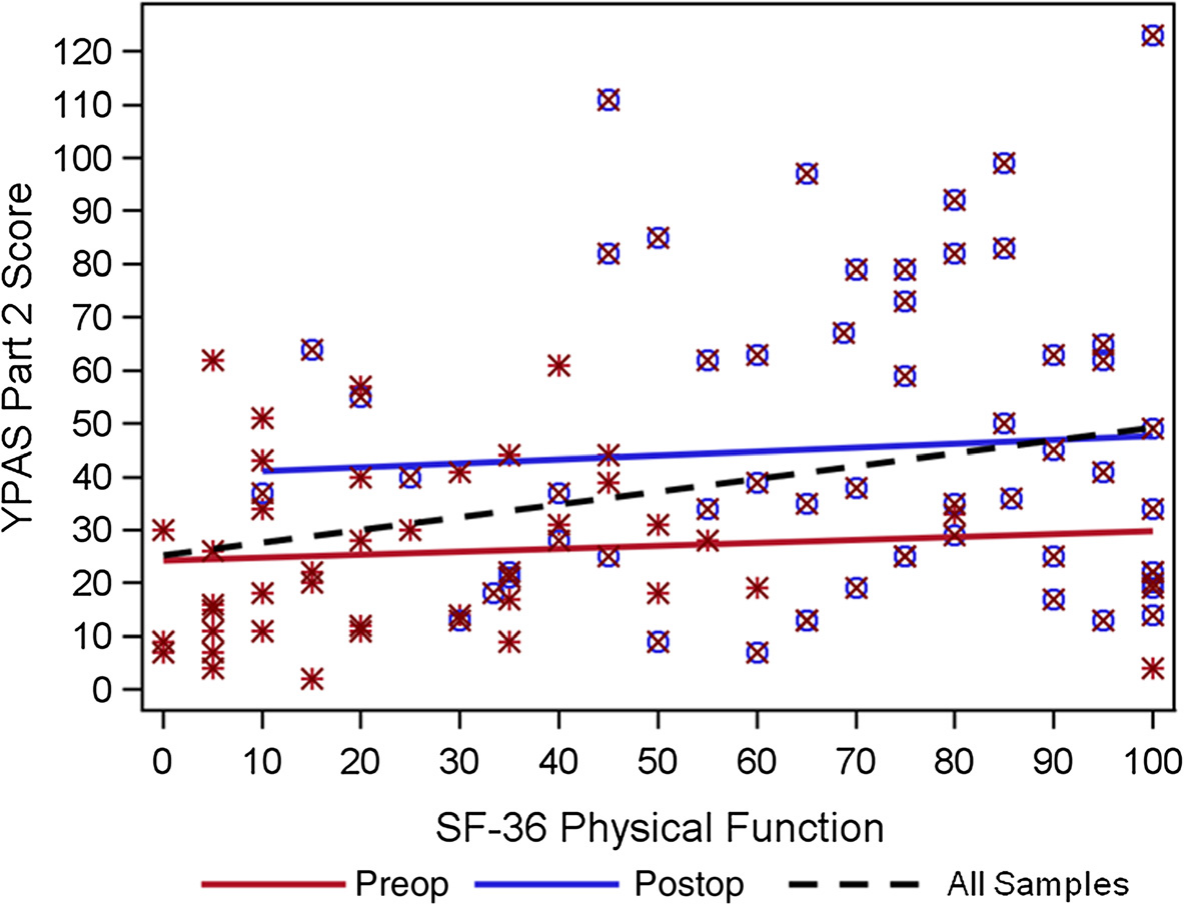
Scatterplot of SF-36 physical function and YPAS Part II score.

We hypothesized that subjects with greater physical activity would have better functional status and quality of life and fewer painful joints. The bivariate associations testing these hypotheses are shown in Table 2 and demonstrate positive moderate correlations (|r| = 0.15 to 0.34) between the total YPAS Part II ADSI score and better functional status and quality of life and fewer painful joints. In stratified analyses, the associations between YPAS ADSI scores and measures of physical activity, functional status and quality of life were comparable among patients assessed preoperatively and those assessed at postoperative visits. The association between YPAS II ADSI scores and the SF-36 Physical Activity Scale are plotted in Figure 1. The Figure demonstrates the positive correlation between the YPAS II ADSI and SF-36 scores and the considerable scatter.

**Table 2:** Correlations among measures of health status and the YPAS scores in the combined preoperative and follow-up samples (Spearman’s correlation coefficient)

	**YPAS Part 1**	**YPAS Part II**	
	Energy expenditure index	Activity dimensions summary index	Total minutes vigorous activity and walking
WOMAC function, r	-0.07	-0.21	-0.15
(p-value)	(0.49)	(0.03)	(0.15)
SF-36 physical activity, r	0.03	0.29	0.21
(p-value)	(0.74)	(0.004)	(0.04)
EQ-5D utility, r	0.18	0.34	0.24
(p-value)	(0.07)	(0.0007)	(0.02)

WOMAC = Western Ontario Osteoarthritis Index.

**Figure 2:**
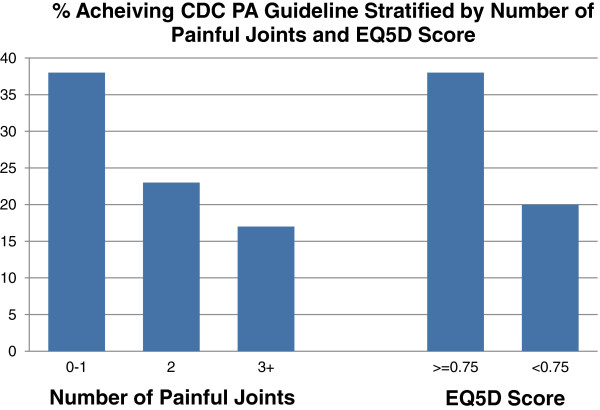
The number of subjects meeting CDC guidelines for physical activity stratified by self-reported number of painful joints and by EQ-5D utility score.

We also posited that patients with fewer painful joints and those with better quality of life (as measured by the EQ-5D) would be more likely to meet the CDC physical activity guidelines than patients reporting more painful joints or worse quality of life. Figure 2 demonstrates that this hypothesis was confirmed. For example, the proportion of subjects meeting the CDC recommendation ranged from 38% of those with just 0 or 1 painful joint to just 17% of those with three or more painful joints (p for trend = 0.23). Thirty-eight percent of the subjects with better quality of life met the CDC recommendation while only 20% of those with lower quality of life met the CDC recommendation (p = 0.07).

The energy expenditure scale in Part I of the YPAS was not associated with any of these measures of functional status or quality of life (WOMAC function scale (r = 0.07, p = 0.49, SF-36 Physical Activity Scale (r = 0.03, p = 0.74) and EQ-5D utility (r = 0.18, p = 0.07).

## Discussion

We used well established methods to develop a Spanish translation of the Yale Physical Activity Survey and then examined the validity of the YPAS in a population of Dominican patients who were about to undergo total knee or hip replacement or who had undergone one of these procedures one to four years previously. All patients were participants in Op Walk Boston, an organization that performs total joint replacement (TJR) in Dominican patients with advanced arthritis who could not otherwise afford the procedures. Patients found the questionnaire acceptable. We documented moderate, positive correlations between the YPAS Part II score -- which measures the extent of vigorous physical activity, walking and stair climbing -- and standard measures of pain, functional status and quality of life. We used the YPAS to calculate the proportion of subjects who met US CDC recommendations for physical activity and documented that patients reporting fewer painful joints or better quality of life were more likely to achieve the recommended levels of physical activity.

Our results compare favorably with previous validations of the YPAS in both English and Spanish-speaking populations. Using an English version, Young [12] found that global indices of the YPAS (specifically, weekly energy expenditure, total time in activity, the summary index and vigorous activity index) correlated with corresponding measures of the Stanford 7-day Physical Activity Recall (r = 0.29-0.51), while Pennathur [27] confirmed moderate test-retest reliability of the YPAS in older Mexican-American adults (e.g. two week retest of the vigorous activity index from Part II YPAS had Spearman r = 0.59). In prior work on Spanish translations of the YPAS, De Abajo [18] found a positive but relatively weak correlation between total time (r = 0.20) and energy expenditure (r = 0.23) recorded by the YPAS and Caltrac accelerometry activity units in Spanish elderly, while Martin [17] found modest correlation between 6-Minute Walk Test and the total time (r = 0.35) and energy expenditure scales (r = 0.38) from the YPAS in a population of elderly Spanish women. Although none of these studies drew upon the same standard measures of physical pain and function as we did here, they nevertheless document reflect consistency in the ability of the YPAS to reflect pain, function and quality of life.

Several aspects of our results bear particular mention. First, the lack of association between the YPAS Part I scale and measures of pain, function and quality of life may reflect the fact that for many patients doing housework is non-discretionary, even if the patient has pain and lower self-rated quality of life. Part I of the YPAS places a heavy emphasis on these home activities, whereas Part II places greater weight on vigorous activity, walking and stairs, which likely are more discretionary and therefore affected by pain, functional status and self-perceived quality of life. Our results are strikingly similar to those of Semanik and colleagues who also found weak correlations between average minutes of moderate or vigorous activity measured with accelerometry and YPAS I measures of total time index (r = 0.05) and energy expenditure index (r = 0.13) [16]. Second, the correlations between the YPAS Part II ADSI score and pain, function and quality of life were weak to moderate, with correlation coefficients in the 0.19-0.34 range. The lack of a strong association between physical status and functional status is consistent with prior studies performed in other populations [17,18,27] and reinforces the conceptual distinction between functional status (what the individual is capable of doing) and physical activity (what they actually do). In fact, the fact the wide variation between physical capacity and physical activity provides the context for public health efforts to encourage patients engage in more physical activity. Finally, the CDC recommendations are used in this study to provide a meaningful threshold of physical activity, but we acknowledge that these recommendations were developed principally for US populations. We feel this is a reasonable substitution, however, given the similarity between the CDC guidelines and the WHO’s global recommendations on physical activity for health for people over 65 years [28].

The strengths of this study include the adherence to established recommendations for translation of health status questionnaires and the availability of a sample with a broad range of functional capacity, with some patients experiencing advanced arthritis just prior to joint replacement, and others experiencing many fewer limitations because they had undergone total joint replacement some years earlier. An important limitation of our work is that it was conducted in a population with advanced arthritis, either prior to or following TJR. Additional work should be done in populations with other conditions and in healthy individuals to further assess validity.

In sum, this translation of the YPAS appears to be well accepted and valid in this initial field test. We suggest further work be done in other conditions and in other Spanish speaking populations, after careful review to ensure that the language used in this version, tailored for the Dominican population, will be understood by other Spanish speaking people in their own countries and the countries where they have immigrated.

## Conclusion

We have used a standardized and rigorous methodology to translate the Yale Physical Activity Survey, and we have demonstrated the validity of the translated instrument. We suggest that other investigators use this instrument in their research on physical activity in subjects from the Dominican Republic. We also suggest that this instrument be used much more broadly among Spanish speaking populations, with attention to potential changes in wording to ensure the instrument is appropriate for distinct Spanish speaking communities.

## Competing interests

The authors declare that they have no competing interests.

## Authors’ contributions

JNK conceived the study, oversaw the translation, drafted much of the manuscript and took responsibility for all components of the study. NNN and MTP performed key aspects of the translation, contributed to study design and revised the manuscript for key intellectual content. YD designed and implemented the analyses and revised the manuscript for key intellectual content. SAB contributed to study design, participated in the translation, drafted sections of the manuscript and revised the manuscript for key intellectual content. DSS, SAE, AS and ESS contributed to the study design, translation, data collection, interpretation of data analyses and revised the manuscript for key intellectual content. JEC contributed to the design of the analytic strategy, oversaw data management and data analysis and revised the manuscript for key intellectual content. All authors read and approved the final manuscript.

## Pre-publication history

The pre-publication history for this paper can be accessed here:

http://www.biomedcentral.com/1471-2474/15/120/prepub

## Supplementary Material

Additional file 1 Spanish translation of the Yale Physical Activity Scale. Click here for file
