# Supplementary material for: Development and validation of a Spanish translation of the Yale activity questionnaire
Source: BMC Musculoskelet Disord. 2014 Apr 7;15:120. doi: 10.1186/1471-2474-15-120 (PMC4027998; doi:10.1186/1471-2474-15-120)
Supplement: Additional file 1 — Spanish translation of the Yale Physical Activity Scale. [file 1471-2474-15-120-S1.pdf]

Cuestionario de Actividad Física de Yale

Entrevistador: favor documente fecha y hora de la cita	Fecha:	Hora:

Existe muy poca información acerca de los patrones de actividad física en individuos de edad avanzada, por lo cual, se está llevando a cabo este estudio para medir cuán activas son las personas de edad avanzada. Adicionalmente, nos gustaría determinar las actividades en las cuales participan las personas de edad avanzada con más frecuencia.

Le voy a hacer unas preguntas acerca de las actividades que usualmente se llevan a cabo, como parte de su rutina diaria y también por placer. No existen respuestas correctas o incorrectas. Su nombre no será utilizado en los resultados, de manera que la información que usted comparta conmigo permanecerá confidencial y será utilizada solo para fines de investigación.

Al dar de su tiempo para llenar los cuestionarios, nos permitirá entender mejor los problemas de salud que afligen a algunas personas mayores. Esta información nos puede ayudar a idear formas en las que podemos mejorar la salud y el bien estar de las personas mayores.

Código: ______________________________

Nombre: ______________________________

Dirección 1: ______________________________

Dirección 2: ______________________________

Número Telefónico: ______________________________

Fecha de nacimiento: ______________________________

Edad: ______________________________

Fecha de la entrevista: ______________________________

Parte Uno

Entrevistador: (favor leer al participante): Nos interesa aprender acerca del tipo de actividades que forman parte de su rutina diaria. Le voy a mostrar listas de tipos de actividades físicas comunes. Por favor, dígame cuanto tiempo (en minutos u horas) dedicó a ellas durante esta semana pasada.

Entrevistador: Muestre al participante la Tarjeta numero 1.

**Actividad**	**Tiempo** **Horas**	**Minutos**	**Código de intensidad**
**Trabajo**			
Compras (ej., supermercado, ropa)	_________	_________	3.5
Subiendo escaleras con una carga.	________	_________	8.5
Lavando:	_________	_________	
Descargando/cargando máquina,	_________	_________	3.0
colgando, doblando,	_________	_________	
lavando ropa a mano	_________	_________	4.0
Trabajo de casa ligero: organizando, despolvando, barriendo, recogiendo basura en el hogar, puliendo, planchando.	_________	_________	3.0
Trabajo de casa pesado: barriendo, trapeando, fregando piso y paredes, moviendo muebles, cajas o zafacones de basura.	_________	_________	4.5
Preparación de comida: cortando, mezclando, buscando los alimentos y útiles para cocinar.	_________	_________	2.5
Servicio de comida: poniendo la mesa, cargando comida, sirviendo comida	_________	_________	2.5
Limpieza: recogiendo la mesa, lavando / secando platos, guardando platos.	_________	_________	2.5
Reparaciones del hogar ligeras: reparación de aparatos pequeños, mantenimiento del hogar ligero / reparaciones.	_________	_________	3.0

Trabajo	Horas	Minutos	Código de intensidad
Reparaciones del hogar pesadas: pintando, carpintería, lavando/puliendo vehículo.	__________	__________	5.5
Otro: ________________________________	__________	__________	______

Trabajo en el patio	Horas	Minutos	Código de intensidad
Jardinería, podando, plantando, deshierbando, cavando, surcando	__________	__________	4.5
Cortando césped (solo si caminando)	__________	__________	4.5
Aclarando caminos/marquesinas: barriendo, paleando, rastrillando	__________	__________	5.0
Otro: _______________________________	__________	__________	______

Cuidando	Horas	Minutos	Código de intensidad
Personas mayores o discapacitadas (cargando, empujando silla de ruedas)	__________	__________	5.5
Niños (haciendo fuerza, cargando, empujando coche)	__________	__________	4.0

Ejercicio	Horas	Minutos	Código de intensidad
Caminando enérgicamente	__________	__________	6.0
Ejercicios de piscina, estiramientos, yoga	__________	__________	3.0
Calistenia vigorosa, aeróbicos	__________	__________	6.0
Ciclismo	__________	__________	6.0
Nadando (solo vueltas)	__________	__________	6.0
Otro: ___________________________	__________	__________	______

Recreo	Horas	Minutos	Código de intensidad
Caminando sin prisa/lentamente	_________	_________	3.5
Costura: tejiendo, cosiendo, punto de cruz, etc.	_________	_________	1.5
Bailando: en grupo, en pareja, etc.	_________	_________	5.5
Pescado	_________	_________	3.0
Nadando, beisbol	_________	_________	5.0
Balancesto	_________	_________	7.0
Billar	_________	_________	2.5
Otro: _________________________	_________	_________	______

Parte Dos

Entrevistador: *favor leer al sujeto:*

Ahora me gustaría preguntarle acerca de ciertos tipos de actividades que ha realizado durante el último mes. Le voy a preguntar acerca de la cantidad de actividad vigorosa, caminatas lentas, tiempo sentado(a), parado(a) y algunas otras cosas, que hace regularmente.

1. ¿Aproximadamente, cuántas veces en el mes participó de actividades vigorosas, que duraron más de **10 minutos** y causaron aumentos importantes en su respiración, ritmo cardíaco, y cansancio de las piernas, <u>o</u> le llevaron a sudar?

Puntuación: Ninguna vez (pase a la pregunta 3)	0
1-3 veces por mes	1
1-2 veces por semana	2
3-4 veces por semana	3
5 + veces por semana	4
Prefirió no contestar	7
No sabe	8

Puntuación por frecuencia _________

2. ¿Aproximadamente cuanto tiempo le dedica cada vez a estas actividades vigorosas?<br><br>No aplica 0<br><br>10-30 minutos 1<br><br>31-60 minutos 2<br><br>60 + minutos 3<br><br>Prefirió no contestar 7<br><br>No sabe 8<br><br>Puntuación por duración ________<br><br>Peso 5
**Puntuación índice por actividad vigorosa:**<br><br>Puntuación por frecuencia ______ X Puntuación por duración _______ X Peso _____ = ______<br><br>(Respuestas 7 u 8 son marcadas como ausentes)

3. Piense en las veces que ha caminado en el último mes. ¿Aproximadamente, cuántas veces en el mes caminó **por lo menos 10 minutos** o más **sin detenerse,** sin que la actividad fuese lo suficientemente agotadora como para causarle aumentos importantes en su respiración, ritmo cardíaco, o cansancio de las piernas, o que le llevara a sudar?<br><br>Puntuación: Ninguna vez (pase a la pregunta 5) 0<br><br>1-3 veces por mes 1<br><br>1-2 veces por semana 2<br><br>3-4 veces por semana 3<br><br>5 + veces por semana 4<br><br>Prefirió no contestar 7<br><br>No sabe 8<br><br>Puntuación por frecuencia _________

4. Cuando se encontraba caminando de esta forma, ¿por cuántos minutos lo hacía?

No aplica	0
10-30 minutos	1
31-60 minutos	2
60 + minutos	3
Prefirió no contestar	7
No sabe	8

Puntuación por duración ________

Peso: 4

Puntuación índice por caminatas sin prisa:

Puntuación por frecuencia ______ X Puntuación por duración ______ X Peso ______ = ______

(Respuestas 7 u 8 son marcadas como ausentes)

5. ¿Aproximadamente, cuántas horas al día pasa haciendo cosas que le requieren estar de pie y moviéndose? Favor reporte solo ocasiones en las que está **realmente moviéndose**.

Ninguna	0
Menos de una hora al día	1
1-3 horas al día	2
3-5 horas al día	3
5-7 horas al día	4
Más de 7 horas al día	5
Prefirió no contestar	7
No sabe	8

Puntuación por movimiento _________

Peso 3

Puntuación índice por movimiento:

Puntuación de movimiento ______ X Peso ______ = ______

(Respuestas 7 u 8 son marcadas como ausentes)

6. Piense en la cantidad de tiempo que pasó parado(a) o moviéndose sobre sus pies durante un día común en el último mes. ¿Aproximadamente, cuántas horas al día pasa **parado(a)**?

Ninguna	0
Menos de una hora al día	1
1-3 horas al día	2
3-5 horas al día	3
5-7 horas al día	4
Más de 7 horas al día	5
Prefirió no contestar	7
No sabe	8

Puntuación por tiempo parado(a) _________

Peso 3

Puntuación índice por tiempo parado(a):

Puntuación por tiempo parado(a) ______ X Peso ______ = ______

(Respuestas 7 u 8 son marcadas como ausentes)

7. ¿Aproximadamente, cuántas horas al día pasaba sentado(a) en un día común durante el mes pasado?

Ninguna	0
Menos de tres horas al día	1
3-6 horas al día	2
6-8 horas al día	3
Más de 8 horas al día	5
Prefirió no contestar	7
No sabe	8

Puntuación por estar sentado(a) _________

Peso 1

Puntuación índice por tiempo sentado(a):

Puntuación por tiempo sentado(a) ______ X Peso ______ = ______

(Respuestas 7 u 8 son marcadas como ausentes)

8. ¿Aproximadamente, cuántos pisos sube andando por escaleras **cada** día? (1 piso = 10 escalones)

____

9. Por favor, compare la cantidad de actividad física que hace durante otras temporadas del año con la cantidad de actividad que acaba de reportar como representativa del mes pasado. Por ejemplo, durante el verano, ¿usted hace más o menos actividad física de la que ha reportado en el último mes?

Entrevistador: *favor circular la puntuación adecuada correspondiente a cada temporada.*

	Mucha más	Poquito más	Igual	Poquito menos	Mucha menos	No sabe
Primavera	1.30	1.15	1.00	0.85	0.70	.
Verano	1.30	1.15	1.00	0.85	0.70	.
Otoño	1.30	1.15	1.00	0.85	0.70	.
Invierno	1.30	1.15	1.00	0.85	0.70	.

Puntuación con ajuste temporal: = suma de todas temporadas / 4 = ______________

Entrevistador: favor documentar el tiempo: _____ _____

Hora Minuto
